# Skin Punch as a Potential Diagnostic Tool for Peripheral Neuropathies of Dogs: Set up of an Indirect Immunofluorescence Protocol on Formalin-Fixed Paraffin-Embedded (FFPE) Biopsy

**DOI:** 10.3390/vetsci12040291

**Published:** 2025-03-21

**Authors:** Maria Teresa Mandara, Simona Arcaro, Ilaria Porcellato, Giuseppe Giglia

**Affiliations:** Department of Veterinary Medicine, University of Perugia, 06126 Perugia, Italy; simonarcaro@gmail.com (S.A.); ilariaporcellatodvm@gmail.com (I.P.); giglia.giuseppe.93@gmail.com (G.G.)

**Keywords:** dog, immunofluorescence, paraffin block, peripheral neuropathy, skin biopsy

## Abstract

In veterinary medicine, the diagnosis of peripheral neuropathies is currently performed using semithin sections or nerve fiber teasing from nerve biopsy. However, these methods actually fail to identify more specific length-dependent and somatosensitive neuropathies. In humans, skin punch biopsy is used to diagnose the latter, through the identification and count of intraepidermal nerve fibers (IENFs) crossing the dermal–epidermal junction, with indirect immunofluorescence (IIF). However, the current need for frozen samples for this technique limits its routine application in clinical practice. In this study, we set up an IIF protocol to identify IENFs in dogs’ skin punch biopsies. Six tests were performed on canine formalin-fixed paraffin-embedded (FFPE) 8 mm skin punches, using an antibody anti-PGP9.5, also known as ubiquitin carboxyl-terminal hydrolase-1. Three parameters were checked: (1) the effectiveness of the co-localization immunoreaction, (2) the thickness of sections, and (3) the magnification for image acquisition. The best IIF results in terms of the sharpness of fiber visualization and the possibility to count them were obtained with 10 µm sections, with a high-power field (×40), without co-localization for nuclei and epithelial structures. Reference data concerning the IENF density of different skin regions in healthy animals of different ages remain to be defined for future diagnostic applications.

## Simple Summary

In dogs, the use of semithin sections or nerve fiber teasing from a nerve biopsy in the diagnosis of peripheral neuropathies fails to identify more specific length-dependent and somatosensitive neuropathies. In humans, skin punch biopsy is used for this purpose, through the identification and count of intraepidermal nerve fibers (IENFs) with indirect immunofluorescence (IIF). However, the current need for frozen samples for this technique limits its routine application in clinical practice. In this study, we set up an IIF protocol to identify IENFs in dogs’ 8 mm skin punches using common formalin-fixed paraffin-embedded sections and the antibody anti-PGP9.5. (1) The effectiveness of the co-localization immunoreaction, (2) the thickness of sections, and (3) the magnification for image acquisition were checked. The best IIF results in terms of sharpness of fiber visualization and possibility to count them were obtained with 10 µm sections, at a ×40 magnification, without co-localization for nuclei and epithelial structures.

## 1. Introduction

The skin is a highly complex organ, containing numerous fine peripheral nervous elements involved in stimuli perception, thermoregulation, and transpiration. For these reasons, specific innervation is required, from both the somatic nervous system (SNS) and the autonomic nervous system (ANS) [1]. Cutaneous nerve fibers of the SNS receive exogenous stimuli, such as tactile, thermal, and noxious stimuli. Some of these fibers are associated with receptors of different structures and complexity, while others are free-ending nerve fibers [1].

**Figure 1:**
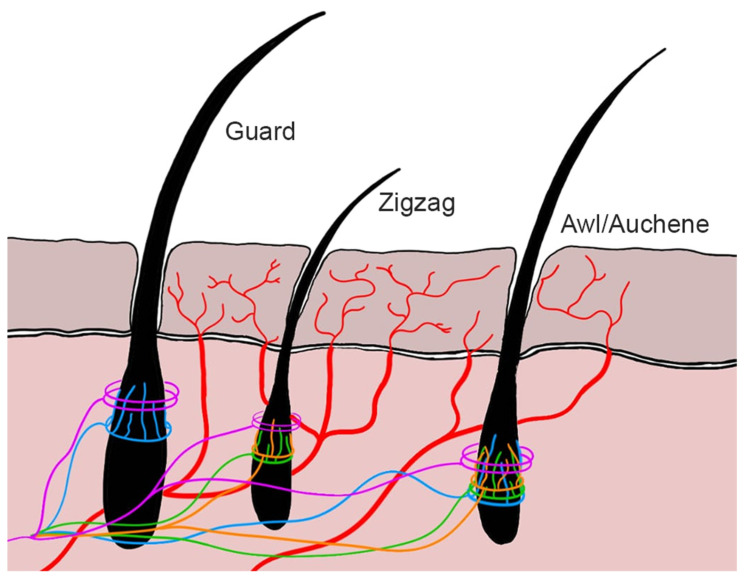
Cutaneous innervation. Dark-brown pink. Epidermis. Light-brown pink: Dermis. Black: hair follicles and hair. Red: free nerve endings. Violet: circumferential lanceolate endings. Orange: longitudinal lanceolate endings (C). Green: longitudinal lanceolate endings (Aδ-LTMR). Blue: longitudinal lanceolate endings (Aδ-RA-LTMR). RA = rapidly adapting; LTMR = low-threshold mechanoreceptors).

Type Aδ and type C nerve fibers play a crucial role in skin receptors along with a number of extremely specialized mechanoreceptors lodging in hairy or hairless skin. They consist in free-ending nerve fibers that perceive noxious stimuli [2] (Figure 1). Aδ nociceptors are myelinated nerve fibers, so they transmit impulses faster than C nociceptors which are instead non-myelinated fibers. For this reason, Aδ fibers are responsible for the first painful pinprick sensation. They are divided into high-threshold mechanoreceptors type I and type II [2].

C nociceptors are non-myelinated nerve fibers transmitting secondary, generally dull, and more diffuse pain. They are polymodal receptors and represent 90% of cutaneous nociceptors; just a small population of them are stimulated by thermic stimuli [2]. Similarly to Aδ nociceptors, type C nociceptors can be divided in high-threshold mechanoreceptors (noxious mechanical and thermal stimuli and non-noxious warm/cold thermal stimuli and chemical stimuli) and low-threshold mechanoreceptors (pleasant mechanical stimuli) [1].

Cutaneous nerve fibers of the ANS consist of type B and efferent type C fibers. These fibers innervate blood vessels, glands, and arrector pili muscles [1].

In the past 20 years, peripheral neuropathies of dogs have been increasingly included in specialized diagnostic investigations. Currently, precise morphological studies of lesions are performed using semithin sections or nerve fiber teasing from nerve biopsy [3]. However, nerve biopsy is an invasive and relatively expensive diagnostic tool reserved for specialized neurologists.

While semithin sections and nerve fiber teasing provide an accurate morphological study of axons and myelin [4], they actually fail to identify more specific length-dependent and somatosensitive neuropathies [5,6,7]. In humans, skin biopsy is an essential tool used for this purpose [8,9], based on the identification and count of nerve fibers crossing dermal–epidermal junction, called intraepidermal nerve fibers (IENFs), with standardized indirect immunofluorescence (IIF) protocols [10,11,12,13]. However, the need for frozen samples limits the routinary application of this technique in clinical practice. In addition, contrary to humans, the dense haircoat skin of dogs makes this potential diagnostic tool harder to use than you might expect and paradoxically less viable. Currently only one study using indirect immunofluorescence on canine skin biopsies has been published, defining changes in IENF density in atopic animals [14].

The aim of this study was to optimize an IENF-staining protocol for indirect immunofluorescence on formalin-fixed paraffin-embedded (FFPE) skin biopsies by developing a minimally invasive technique to perfect and complete the diagnosis of peripheral neuropathies in veterinary medicine. In addition, we aimed to evaluate the possibility of counting IENFs as a starting point for future defined reference ranges in healthy dogs.

## 2. Materials and Methods

After hair trimming, skin biopsies were collected with 8 mm punches from 4 dogs referred to our department for autopsy, after obtaining owner consent. To test protocols capable of detecting the IENFs, dogs not affected by peripheral neuropathies nor metabolic diseases were selected in this study. The animals were an 18-month-old female English setter dog, 2-year-old female Chihuahua dog, 15-year-old male mixed-breed dog, and 3-month-old female Maremma sheepdog. An acute severe hemorrhagic enteropathy was the cause of death for all of them, except for the Chihuahua dog submitted to euthanasia with an epileptic status associated with uncompensated hydrocephalus. Sampling was obtained no more than 12 h after death, with the dogs stored in the refrigerator. Skin biopsies were immediately fixed in 10% neutral-buffered formalin for 4 days and subsequently paraffin-embedded following routinary protocols.

### 2.1. Indirect Immunofluorescence (IIF) 

For IIF, a total of six tests were performed. The first three tests were to set up the IIF protocol for FFPE skin biopsy. They were carried out on skin biopsies performed on a paracervical site (lateral aspect of the neck overlying the larynx), being one of the reference anatomical sites previously investigated in dogs [5], and corresponding to the paraspinal region as in the guidelines for human peripheral neuropathy studies [15]. The first test was randomly performed in the 18-month-old female English setter dog, while both the second and the third tests were performed in the 2-year-old female Chihuahua dog. The last three tests were performed on skin biopsies obtained from the hind limb, at proximal level (lateral aspect of the thigh), as the main anatomical site commonly used to test peripheral neuropathies in dogs and humans [15]. They were performed in dogs of three different age categories: a 3-month-old puppy female Maremma sheepdog, a 2-year-old female Chihuahua dog, and an aged 15-year-old male mixed-breed dog.

Intraepidermal nerve fibers were identified using an anti-human PGP 9.5 rabbit polyclonal antibody (1:450; GeneTex, Milan, Italy). PGP 9.5, also known as ubiquitin carboxyl-terminal hydrolase-1 (UCH-L1), is a cytosolic ubiquitin carboxyl-terminal hydrolase expressed by nervous and neuroendocrine cells [16], for which a similar pattern of expression has been reported in dogs [17]. In skin biopsies, the antibody recognizes dermal nerve bundles and both peptidergic and non-peptidergic epidermal nerve profiles [18]. Spinal cords were used both as positive and negative controls. The negative control procedure was performed following the same protocol used for the actual test but omitting the primary antibody.

To highlight epithelia (epidermis, glands, and hair follicles), a mouse monoclonal antibody anti-human cytokeratin (CK) (1:200; AE1/AE3, Agilent, Milan, Italy) was used. Cell nuclei were marked with DAPI (Abcam, Milan, Italy). The processed sections were observed with an epifluorescence microscope (Olympus BX51, Olympus U-RL-T fluorescent lamp, Tokyo, Japan). For the final IENF count, pictures were taken with NIS-Elements D software (5.10.00 version, Nikon, Tokyo, Japan) from five consecutive fields per each section. Only single IENFs crossing the dermal–epidermal junction were considered, while secondary branching and fragments were excluded from quantification [19].

After deparaffinization and rehydration, antigen retrieval was performed by microwaving the slides in Tris-EDTA buffer solution (10 mmol/L Tris Base, 1 mmol/L EDTA, pH 9.0) for 2 min at a high temperature (900 W), followed by 20 min at a low temperature (200 W). Endogenous peroxidase was blocked with 3% hydrogen peroxide in methanol for 45 min. Then, the first primary antibody anti-CK was incubated at 4 °C overnight in a humidity chamber. Green fluorochrome (1:200; Donkey pAb anti-Mouse Alexa Fluor^®^ 488; Abcam, Milan, Italy) was applied as the first secondary antibody for 1.5 h at room temperature. The second primary antibody anti-PGP9.5 was then incubated at 4 °C for 2 h in a humid chamber. Red fluorochrome (1:200; Goat pAb anti-Rabbit Alexa Fluor^®^ 594; Abcam, Milan, Italy) was used as a second secondary antibody and incubated for 1.5 h at room temperature. Finally, the specimens were mounted with DAPI (blue fluorochrome) and cover-slipped.

In the first three tests, sections of 5 μm and 30 μm thicknesses were compared. They were performed in sequence, each of them coming from the previous to optimize the next. The efficacy of the pictures showing the co-localization of fluorescence obtained by merging three photos (one for each fluorochrome) in the same field (protocol 1) compared to the pictures without co-localization (protocol 2) and the magnification for the best traceability of IENFs were also tested. The last three tests were performed on the 2-year-old female Chihuahua dog, 15-year-old male mixed-breed dog, and 3-month-old female Maremma sheepdog. Sections of 5 μm and 10 μm were compared and pictures without the co-localization of fluorescence were obtained (Protocol 2). IENFs were counted in five consecutive fields at x40 magnification. Methods applied to IIF are summarized in Table 1.

### 2.2. Immunohistochemistry (IHC)

Additional 5 μm thickness FFPE skin sections from the proximal hind limb were submitted to immunohistochemistry (IHC) with rabbit polyclonal antibody anti-human PGP9.5 (1:450; GenTex, Milan, Italy). Immunohistochemistry tests were performed on the hind limb skin biopsies in parallel with the 4, 5, and 6 IIF tests, which summarized the adjustments of the previous tests.

After deparaffinization and rehydration, antigen retrieval was performed using a microwave for 2 min at a high temperature (900 W), followed by 20 min at a low temperature (200 W) in Tris-EDA buffer solution (10 mmol/L Tris base, 1 mmol/L EDTA, Ph 9.0). Endogenous peroxidase was blocked using 3% hydrogen peroxide in water for 5 min at room temperature. Afterward, the slides were covered with the primary antibody for 1 h in a humidified chamber at room temperature. Immunoreactivity was revealed using the avidin–biotin method with aminoethyl-carbazole substrate (AEC Substrate kit, Abcam, Milan, Italy). Carazzi’s hematoxylin was used as the counterstain.

On IHC samples, IENFs were counted in five consecutive fields per section with direct observation at a high magnification (×40). Again, only the single IENFs crossing the dermal–epidermal junction were considered, while secondary branching and fragments were excluded from quantification (Lauria et al., 2005 [19]).

## 3. Results

### 3.1. Indirect Immunofluorescence (IIF)

All tests showed the expected IIF reaction. Green fluorescence labeled CK-positive epithelial structures, whereas red fluorescence identified PGP9.5-positive IENFs and nerve fibers associated with blood vessels, glands, and hair follicles. DAPI fluorescence (blue) labeled cell nuclei.

#### 3.1.1. Test 1

**Figure 2:**
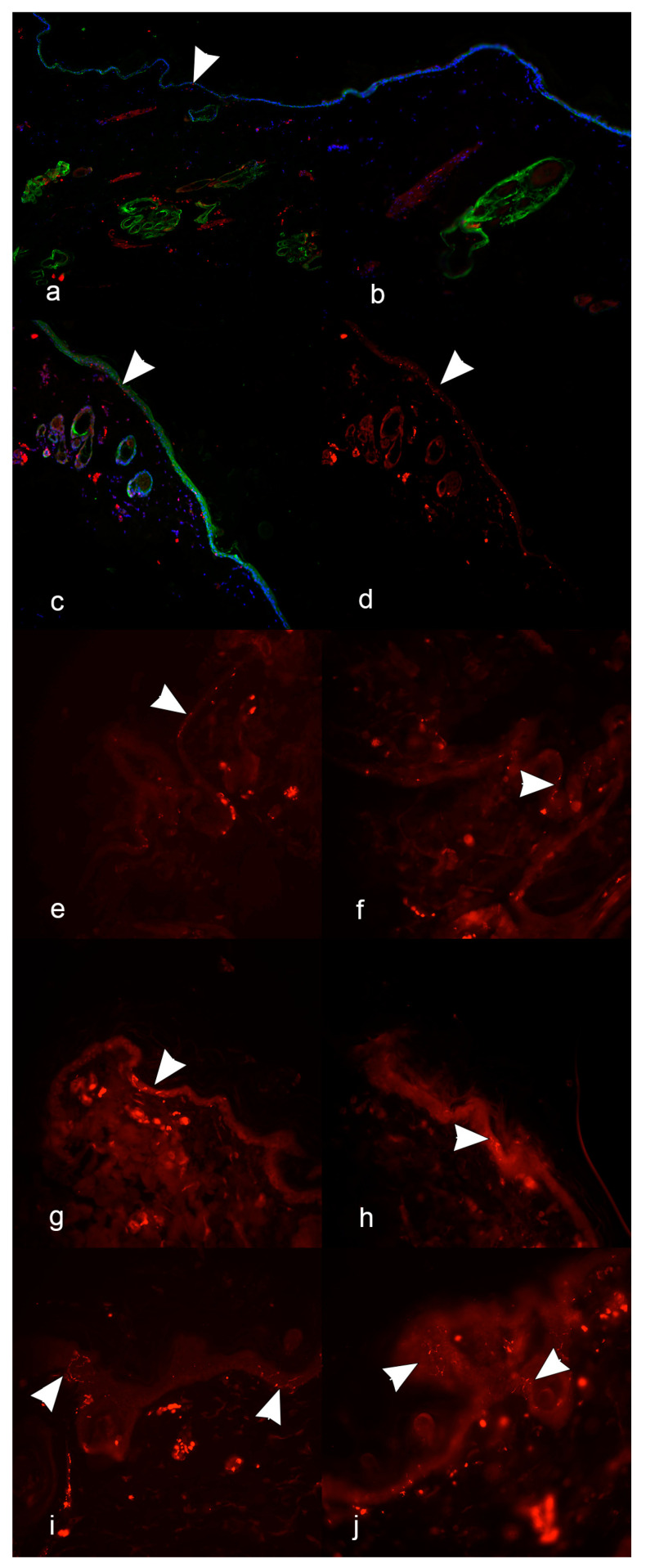
Indirect immunofluorescence on skin biopsies and evidence of intraepidermal nerve fibers (IENFs) in the different tests. IENFs are marked with arrow heads as a red line, or, in less distinct reactions, as a red point. In (**a**–**c**), cell nuclei are marked with DAPI (blue fluorochrome), and the epithelial elements are marked with antibody anti-cytokeratin (green fluorochrome). Paracervical site (**a**–**d**). (**a**) Test 1; protocol 1 (co-localization); 5 μm section (×10). (**b**) Test 2; protocol 1 (co-localization); 5 μm section (×20). (**c**) Test 3; protocol 1 (co-localization); 5 μm section (×40). (**d**) Test 3; protocol 2 (without co-localization); 5 μm section (×40). Proximal hind limb (**e**–**j**). (**e**) Test 4; protocol 2 (without co-localization); 5 μm section (×40). (**f**) Test 4; protocol 2 (without co-localization); 10 μm section (×40). (**g**) Test 5; protocol 2 (without co-localization); 5 μm section (×40). (**h**) Test 6; protocol 2 (without co-localization); 10 μm section (×40). (**i**) Test 6; protocol 2 (without co-localization); 5 μm section (×40). (**j**) Test 6; protocol 2 (without co-localization); 10 μm section (×40). (Anti-human PGP 9.5 rabbit polyclonal antibody).

Paracervical site. Eighteen-month-old female English setter dog. Subcutaneous nervous fascicles, nerve fibers associated with glands and blood vessels (red IF), and epidermis (green IF) were clearly recognized at a ×10 magnification, in a 5 μm section processed with protocol 1 (co-localization), while IENFs were not distinctly identified and their count was omitted (Figure 2a).

#### 3.1.2. Test 2

Paracervical site. Two-year-old female Chihuahua breed dog. IENFs consisting of red free linear ending nerve fibers perpendicular to dermal–epidermal junction were hardly identified with protocol 1 (co-localization) at a ×20 magnification, in a 30 μm section as in the 5 μm section. At this magnification, the crossing of the dermal–epidermal junction by the free-ending nerve fibers was not traceable (Figure 2b).

#### 3.1.3. Test 3

Paracervical site. Two-year-old female Chihuahua breed dog. A straightforward identification of IENFs was obtained in the 5 μm section more than in the 30 μm section, at the ×40 magnification more than at ×20, excluding co-localization (protocol 2). IENFs were linear or just sinuous, running perpendicularly to the basement membrane (Figure 2c,d). Their count was omitted.

#### 3.1.4. Test 4, 5, and 6

Proximal hind limb. In the 2-year-old female, Chihuahua dog (test 4), seven and eleven IENFs were counted in the 5 μm and 10 μm sections, respectively (Figure 2e,f). The IENFs ran as isolated fibers or aggregated in small clusters. In the 15-year-old male mixed-breed dog (test 5), four and three IENFs were counted in the 5 μm and 10 μm sections, respectively (Figure 2g,h). In the 3-month-old female Maremma sheepdog (test 6), 32 and 34 IENFs were counted in the 5 μm and 10 μm sections, respectively (Figure 2i,j).

All the IENF counts are summarized in Table 1.

### 3.2. Immunohistochemistry (IHC)

**Figure 3:**
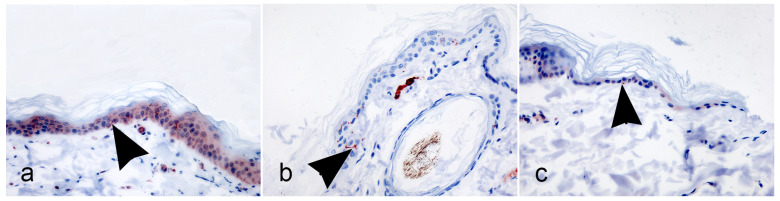
Immnunohistochemistry on skin biopsies performed from proximal hind limb. Intraepidermal nerve fibers (IENFs) are marked with arrow heads. (**a**) Three-month-old dog. A clear linear immunoreaction marks IENFs. (**b**) Two-year-old dog. The complete lack of non-specific background staining in the epidermis clearly marks the IENFs, again as a linear immunoreaction. (**c**) Fifteen-year-old dog. Punctiform immunoreaction less distinctly marks IENFs; (×40). (Anti-human PGP 9.5 rabbit polyclonal antibody; Carazzi’s haematoxylin as a nuclear counterstain).

Skin biopsy was performed on the proximal hind limb (thigh), in parallel to the previous 4, 5, and 6 IIF tests. In all the sections, a marked reaction was observed for IENFs, as well as for periglandular, perifollicular, and perivascular fibers. The immunoreaction showed linear or granular PGP9.5 expression patterns, consisting of clearly distinct and poorly visible IENFs, respectively. IENFs for which crossing dermal–epidermal junction was lost were not counted. Finally, a total of six, twelve, and two IENFs/five HPFs were counted in the 3-month-old Maremma Sheepdog, the 2-year-old Chihuahua, and the 15-year-old mixed breed dog, respectively. (Figure 3a–c) (Table 1).

## 4. Discussion

Peripheral neuropathies and polyneuropathies are common clinical challenges in canine neurology. Due to their non-specific signs, clinical diagnosis needs to be supported by laboratory tests (blood and urine exams), functional studies (nerve electrostimulation), and morphological studies [3]. For this group of neurological diseases, the peripheral nerve biopsy is currently the diagnostic gold standard.

Contrarily to veterinary medicine, since the nineties the cutaneous nerve biopsy has gained widespread use in the diagnosis of human peripheral neuropathies [7], with special respect to length-dependent and somatosensory small fiber neuropathies [19,20]. Skin biopsy is mainly used to identify small caliber nerve fibers, including non-myelinated IENFs (type C nerve fibers), myelinated cutaneous nerve fibers (type Aδ nerve fibers), and autonomic nerve fibers [19]. This technique plays an essential role in this context since these types of fibers are not detectable with traditional nervous conduction studies [7]. Unfortunately, the current protocols consisting of IIF on frozen samples limit routinary application in clinical practice. Moreover, the dense haircoat of dogs calls for the optimization of nerve-staining protocols to best improve the quality of FFPE skin sections.

In this study, we planned to use FFPE samples to set up an IIF protocol allowing neurologists to collect, fix, and submit a biopsy sample to the pathology lab similarly to other specimens commonly submitted for histological examination.

In veterinary medicine, de Medeiros and colleagues [5] were the first to establish an immunohistochemistry protocol to identify cutaneous nerve fibers in canine skin biopsy specimens. The authors followed human guidelines [15], using frozen samples previously fixed with Zamboni’s solution [5], supported by the evidence that frozen tissues are less subject to IENF fragmentation when compared to formalin fixation [21]. They analyzed 20 μm thick sections and marked IENFs through immunohistochemistry staining against PGP9.5 (ubiquitin C-terminal hydrolase) [5]. Different anatomical sites were sampled for each dog.

More recently, Laprais and colleagues [14] analyzed 30 μm FFPE sections from skin biopsies with indirect immunofluorescence staining against beta-3 tubulin. The authors defined IENFs density in atopic dogs compared to healthy animals. Unfortunately, in this study, the samples had been collected over two decades from different geographical locations resulting in discrepancies in tissue fixation methods. Moreover, the duration of formaldehyde fixation among samples was not standardized, introducing further bias.

In the absence of a standardized protocol for the identification of IENFs in dogs, we worked to set up a protocol to identify IENFs from 8 mm skin punches routinely processed as FFPE samples, supported by the evidence that FFPE samples provide good IENF visualization and do not alter their density [15,21,22]. This method enables clinicians to collect skin samples and send them to histopathology laboratories, regardless of the geographical area where they work. Furthermore, this method is of simple feasibility, even in non-specialized pathology labs, and the sampling can be easily performed in clinical practice. However, contrary to humans, the dense haircoat skin of dogs causes technical complications that can compromise the quality of results.

In the first three tests, the IIF protocol was verified to check: (1) the efficacy of the co-localization immunoreaction, (2) the thickness of sections, (3) the magnification for image acquisition. In the first two tests, IENFs were marked along with epithelial elements, using a protocol with the co-localization of PGP9.5 and CK immunofluorescence (protocol 1). However, due to the overlap of all the marked structures at image merging, the visualization and identification of IENFs were greatly hampered. Therefore, in the third test, a new IIF protocol without co-localization (protocol 2) was used, with cell nuclei marked with DAPI for optimal section orientation. The choice to avoid co-localization was also supported by the literature and EFNS guidelines recommending using only one antigen (usually PGP9.5) to study IENFs during peripheral neuropathies [7,13,14,15,19].

At the same time, in the first three tests, the thickness of sections was also tested. Starting from the human literature and guidelines reporting the use of paraffin-embedded 3–30 μm sections for immunohistochemistry [18,19], we first compared 5 and 30 μm thick sections. The 5 μm sections gave us better results than the 30 μm sections in terms of fewer artifacts and a higher quality of images, concluding that the 30 μm sections were too thick for IENF identification in FFPE sections. Although good results were obtained by Laprais with the epifluorescence IIF protocol using 30 μm FFPE sections marked with antibody anti-beta 3 tubulin [14], sections from 30 μm to 100 μm were better suited for confocal immunofluorescence techniques [21]. Moreover, thick sections were better suited for frozen tissue embedded in optimal cutting temperature media, after cryoprotection [18,23]. Due to the unsatisfactory results obtained with 30 μm sections, we proceeded to compare 5 μm and 10 μm thick sections. The 10 μm thick sections enabled us to identify IENFs clearer than the 5 μm thick sections, with a well-defined passage across the dermal–epidermal junction. Nevertheless, in the last test (test 6), we obtained an excellent reaction in the 5 μm sections as well, which could still be a valuable alternative.

Regarding image acquisition, consistently with ENFS [15,19], we observed that a ×40 magnification made the identification of IENFs clearer to distinguish from secondary branching and fragments when necessary. Nevertheless, a ×20 magnification is also recommended when using an epifluorescence microscope [15]. Specific suggestions about magnification were not reported in the unique paper published on dogs, so we can conclude that the magnification for capturing well-defined IENFs depends on personal choice [14]. Nevertheless, in agreement with the study from Lauria and colleagues [19], our data confirm that a high magnification (×40) is recommended to identify and count IENFs in sections using bright-field immunohistochemistry or immunofluorescence.

Based on all the adjustments above, in the last three tests, we applied the herein-described IIF protocol to skin biopsy harvested on the proximal hind limb (thigh) as one of the main anatomic reference sites in the diagnosis of peripheral neuropathies. Moreover, encouraged by the good identification of IENFs, we proceeded to count them on five consecutive fields, as a preliminary method compared to the ENFS guidelines, reporting IENF count on the whole length of the section [15,19]. Adopting this counting method, we wanted to be as minimally influenced as possible by artifacts eventually reported in the canine skin section. In all of these tests, the IENF identification was fluently obtained. Although in this study the results pertaining to the number of IENFs must not be considered conclusive, it seems that the 10 μm sections lead to the identification of a slightly higher number of IENFs/five fields compared to 5 μm. If confirmed, this result supports the notion that the section thickness must be taken into consideration in IENFs count [19]. Moreover, although no final conclusions can be deduced by such a limited number of cases, it also seems that the 3-month puppy expressed a number of IENFs much higher than the adult dog (2-year-old) and that this value tended to dramatically reduce in the old dog (15-year-old). This result was not confirmed by IHC, potentially corroborating the IIF as a superior technique compared to IHC in this field of investigation. Actually, counting IENFs in five consecutive fields could lead to dramatic bias. In fact, the patchy nature of the innervation and clustering of nerve fibers is typical for the epidermis, so that in the same section, areas with very high and very low relative densities of nerve endings may be interspersed [5,19]. This is why linear IENF density in at least three non-consecutive sections (IENFs/mm) is strongly recommended [19] and should also be adopted for the final count in dogs [5]. Nevertheless, if confirmed by further studies planned for this purpose, this age-related trend would be of great support in clinical issues related to skin pain perception in different aged animals.

The results obtained with the IIF protocol set up in this study encourage future investigations to support veterinary pathologists and neurologists in the diagnosis of peripheral neuropathies, using a simple, non-invasive FFPE skin biopsy. Nevertheless, in the present study, a number of limitations need to be addressed. First of all, the IIF protocol will be tested and improved on a larger cohort of cases. Moreover, when using IENF counts for diagnostic purposes, reference values should be established for dogs, taking into account the anatomic site, breed, age, and, possibly, sex.

## 5. Conclusions

This study describes a step-by-step protocol set up for the identification of intraepidermal nerve fibers (IENFs) with an indirect immunofluorescence (IIF) technique performed with antibody anti-PGP9.5 on FFPE canine skin biopsy. Keeping in mind the difficulty of processing the dense haircoat of canine skin, we propose the use of 10 μm sections for epifluorescence IIF as a good compromise between too thick and routinary FFPE sections. High magnification is also necessary to better identify IENFs in captured images, as it allows us to differentiate free IENFs crossing the dermal–epidermal junction from the secondary branching and fragments. The potential methodological limitations of this study need to be recognized: (1) the preliminary profile of the results obtained requires working step by step in selecting the best technical options and (2) the low number of processed skin biopsies. A further systematic work-up is necessary to provide reference values of IENF density in different anatomical areas of dogs, exploiting and validating the described method. Surely, the non-invasive and inexpensive approach to skin biopsy [8], showing a lower impact on the patient compared to traditional nerve biopsy, makes this technique promising and easy to use in clinical practice. Its application in the diagnosis of autonomic and sensory neuropathies in dogs sounds like its most intriguing field of application.

## Figures and Tables

**Table 1:** Main differences in indirect immunofluorescence methods applied to each test, and associated IENF count by IIF and IHC.

Test	Dog	Site of Biopsy	Paraffin Sections	IIF Protocol	Magnification at IENF Count	IIF Count	IHC Count
Test 1	1.5-year-old female English setter	Paracervical	5 μm	Protocol 1	×10	o.c.	
Test 2	2-year-old female Chihuahua	Paracervical	5 μm	Protocol 1	×20	o.c.	
			30 μm				
Test 3	2-year-old female Chihuahua	Paracervical	5 μm	Protocol 1	×20 vs. ×40	o.c.	
			30 μm				
Test 4	2-year-old female Chihuahua	Proximal hind limb	5 μm	Protocol 2	×40	7	12
			10 μm			11	
Test 5	15-year-old male mixed-breed	Proximal hind limb	5 μm	Protocol 2	×40	4	2
			10 μm			3	
Test 6	3-month-old female Maremma sheepdog	Proximal hind limb	5 μm	Protocol 2	×40	32	6
			10 μm			34	

IIF = indirect immunofluorescence; IHC = immunohistochemistry; IENF = intraepidermal nerve fiber; o.c. = omitted count.

## Data Availability

Data are available in the manuscript.
